# Development of the Synarcual in the Elephant Sharks (Holocephali; Chondrichthyes): Implications for Vertebral Formation and Fusion

**DOI:** 10.1371/journal.pone.0135138

**Published:** 2015-09-04

**Authors:** Zerina Johanson, Catherine Boisvert, Anton Maksimenko, Peter Currie, Kate Trinajstic

**Affiliations:** 1 Department of Earth Sciences, Natural History Museum, Cromwell Road, London, SW7 5BD, United Kingdom; 2 Australian Regenerative Medicine Institute (ARMI), EMBL Australia Building 75, Level 1 Monash University, Clayton, Victoria, 3800, Australia; 3 Australian Synchrotron, 800 Blackburn Road, Clayton, Victoria, 3168, Australia; 4 Department of Environment and Agriculture, Curtin University, Bentley, Western Australia, 6845, Australia, and Department of Earth and Planetary Sciences, Western Australian Museum, 49 Kew Street, Welshpool, Western Australia, 6106, Australia; University of Alabama at Birmingham, UNITED STATES

## Abstract

The synarcual is a structure incorporating multiple elements of two or more anterior vertebrae of the axial skeleton, forming immediately posterior to the cranium. It has been convergently acquired in the fossil group ‘Placodermi’, in Chondrichthyes (Holocephali, Batoidea), within the teleost group Syngnathiformes, and to varying degrees in a range of mammalian taxa. In addition, cervical vertebral fusion presents as an abnormal pathology in a variety of human disorders. Vertebrae develop from axially arranged somites, so that fusion could result from a failure of somite segmentation early in development, or from later heterotopic development of intervertebral bone or cartilage. Examination of early developmental stages indicates that in the Batoidea and the ‘Placodermi’, individual vertebrae developed normally and only later become incorporated into the synarcual, implying regular somite segmentation and vertebral development. Here we show that in the holocephalan *Callorhinchus milii*, uniform and regular vertebral segmentation also occurs, with anterior individual vertebra developing separately with subsequent fusion into a synarcual. Vertebral elements forming directly behind the synarcual continue to be incorporated into the synarcual through growth. This appears to be a common pattern through the Vertebrata. Research into human disorders, presenting as cervical fusion at birth, focuses on gene misexpression studies in humans and other mammals such as the mouse. However, in chondrichthyans, vertebral fusion represents the normal morphology, moreover, taxa such *Leucoraja* (Batoidea) and *Callorhinchus* (Holocephali) are increasingly used as laboratory animals, and the *Callorhinchus* genome has been sequenced and is available for study. Our observations on synarcual development in three major groups of early jawed vertebrates indicate that fusion involves heterotopic cartilage and perichondral bone/mineralised cartilage developing outside the regular skeleton. We suggest that chondrichthyans have potential as ideal extant models for identifying the genes involved in these processes, for application to human skeletal heterotopic disorders.

## Introduction

The vertebrate axial skeleton is composed of serially repeated dorsal, central and ventral units extending posteriorly from the skull or braincase to the tip of the caudal fin, or tail [1]. These units develop from sclerotomal cells derived from serially repeated somites ([2–3]; see [4] for a broad summary of vertebral development in various groups); the somites themselves derive rostro-caudally from continuous presomitic mesoderm under the influence of a segmentation clock and gene pathways such as Notch, Wnt and Fgf [5–10]. The dorsal, ventral and central vertebral units are incorporated into individual vertebrae, each of these units being controlled by specific genes [3, 9, 11, 12]. Among vertebrates, there is a high degree of variability in the vertebral column (e.g., number of vertebrae present, morphology of the different vertebrae, contribution of the notochord, whether dorsal, ventral and central elements are present or absent), including the degree of vertebral fusion and the location of that fusion within the column.

**Fig 1:**
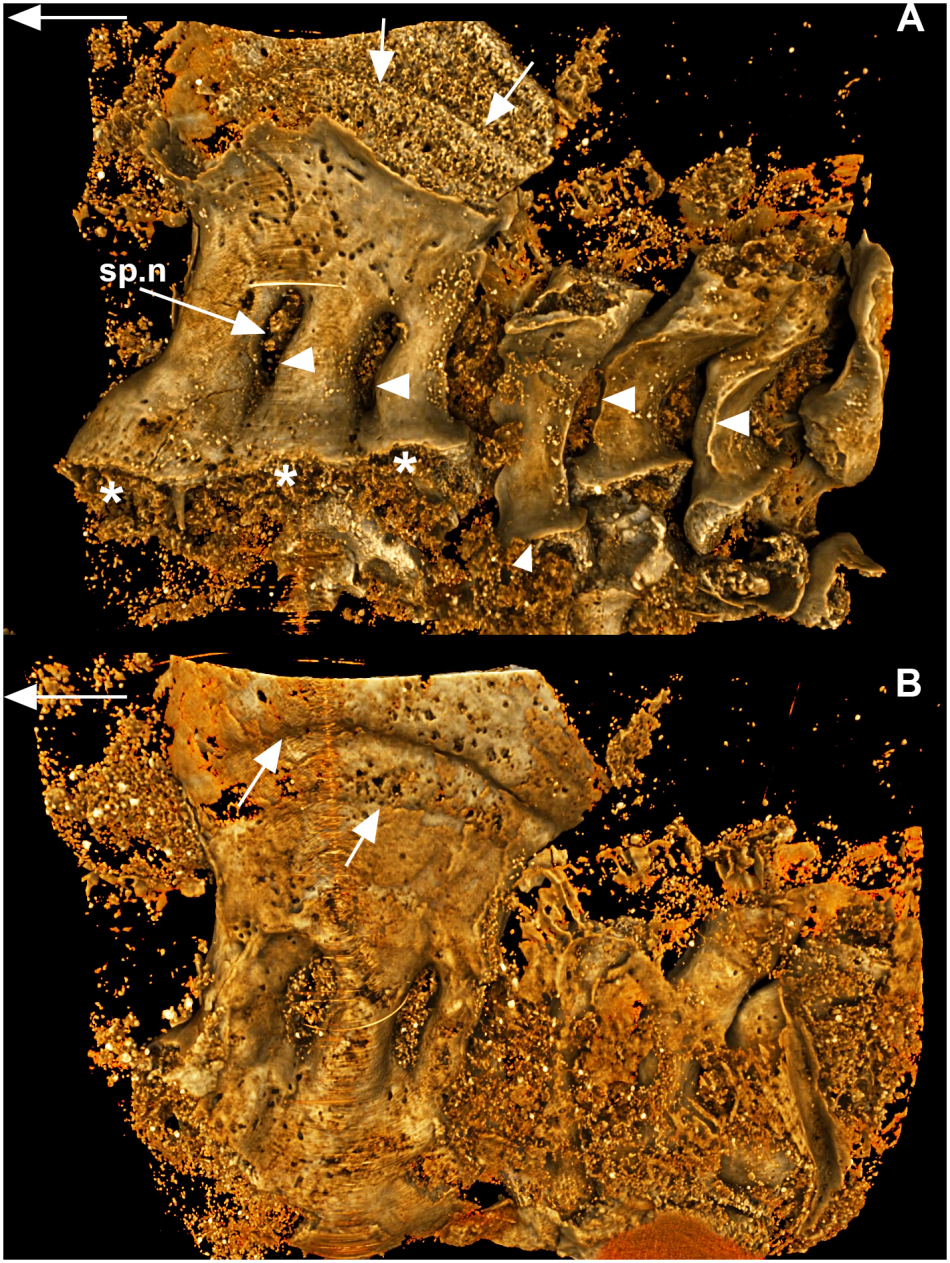
*Compagopiscis croucheri*. (WAM 11.9.1). A, lateral and B, medial views of synarcual comprising three fused vertebral elements (neural arches, spines). Fusion is dorsal and ventral with space between these elements remaining, for the neural spines. The dorsalmost part of the three spines has become enlarged, large white arrows indicate lines of perichondral bone deposition on the developing heterotopic cartilage. Ventrally, the neural arches are also flared (asterisk), particularly the most anterior arch remnant; the third element retains more of the typical circular base shape. Arrowheads indicate preservation of the sharp edges along the incorporated neural spines, as are seen in more posterior separate vertebra of the posterior axial skeleton. Large white arrows indicate anterior direction.

For example, fusion occurs within the vertebral column of teleost fishes, as part of regular development (e.g., the caudal fin) and also as a result of environmental stresses [13–19]. Fusion also occurs to produce the tetrapod sacrum [20–22], all involving fusion of more posterior vertebral elements. With respect to more anterior vertebrae, the notarium in some birds (e.g. Passeriformes) and pterosaurs involves fusion of the thoracic vertebra [21, 23]. Formation of the synarcual involves fusion of the anteriormost, cervical, vertebrae and is found in certain placoderms, a group of fossil fishes from the Silurian to Devonian periods (443.8 +/- 1.5 MYA-358.9 +/-0.4mya; see [24]: figure 1, showing a generalized placoderm; also [25, 26]). A synarcual also occurs in the Holocephali and Batoidea among the Chondrichthyes [24, 27–29] and rarely in teleosts [30–32]. Cervical fusion also occurs in several mammalian taxa, including sloths, manatees, dugongs [33], armadillos [34], whales [35], ricochetal rodents [36], and the marsupial *Didelphis* [37]. In humans, cervical vertebral fusion results from congenital or acquired disease processes. Congenital disorders are either related to a failure of somite segmentation early in development (e.g., Klippel-Feil syndrome), or transformation of tissues surrounding the vertebrae into cartilage and bone (e.g., Fibrodysplasia Ossificans Progressiva) [38–41]. Current research is beginning to identify animal models, such as the salmon, that can be used to explore the causes of this fusion for application to human patients (e.g., [18]). However, vertebral fusion in the salmon is often the result of environmental stresses (an acquired morphology), as noted above; however, the need for ‘evolutionary mutant models’ for human diseases has recently been recognised, where adaptive phenotypes resemble human diseases; for example, the Antarctic icefish lose bone density to increase buoyancy, a potential model for osteoporosis in humans (see Table 1 in [42], also [43]). These ‘evolutionary mutants’ would be complementary to laboratory, or induced mutants, such as the salmon [42].

Because placoderms and chondrichthyans possess fused anterior cervical vertebrae as part of their normal morphology, rather than resulting from environmental stresses, they are potentially ideal models for examining the causes of congenital human cervical disorders. We are beginning to understand the development of the batoid synarcual [26, 27, 29] as well as that of placoderms, the most primitive of the jawed vertebrates [24–26].However, the synarcual of the other major group of chondrichthyans, the Holocephali, has not been studied in detail. We demonstrate below that early ontogenetic stages of the extant holocephalan *Callorhinchus milii* show normal development of axial vertebrae as individual elements, implying that the somites also form individually and normally. Within the adult mineralizing synarcual, the individual vertebrae are retained to some degree and visible, comparable to both placoderms and batoid chondrichthyans. These observations suggest that in most groups of vertebrates with a consistently developing synarcual, fusion occurs after normal vertebral development, more comparable to human cervical disorders where cartilage and bone develop heterotopically in the vertebral column.

## Materials and Methods

### ‘Placodermi’, *Campbellodus decipiens*

A specimen of *Campbellodus decipiens* (Western Australian Museum, WAM 11.9.1) was scanned using synchrotron radiation X-ray tomographic microscopy (SRXTM) at the Imaging and Medical beamline of the Australian Synchrotron, Victoria, Australia (http://www.synchrotron.org.au/index.php/home). The photon energy of the monochromatic X-ray beam was set to 30 keV. Data acquisition included 3600 projections of 2560x2140 pixels each with the pixel size being 6.5 microns and measured resolution 13 microns. Exposure time for a single projection was 1s, resulting in a 1 hour scan for the full sample. The sample to detector distance was 210mm which has enhanced the object visibility due to the inline phase contrast. Data were processed and reconstructed utilising the CTAS package (https://github.com/antonmx/ctas) and rendered with Drishti-2 (https://github.com/AjayLimaye/drishti).

### Holocephali, *Callorhinchus milii*: stages 22, 23, 25, 27, 28, 29, 30

Animals were euthanised via an overdose of tricaine in seawater. We confirm that Monash Animal Services (MAS) specifically approved this study via the awarding of ethics permit MAS/ARMI/2010/01, Monash University, Victoria, Australia. Impregnated females of *C*. *milii* were collected from Western Port bay, Victoria, Australia (DPI permits# RP1003 and RP1112) and housed in aerated, temperature regulated seawater tanks in the Rosebud facility on the Mornington Peninsula, Victoria (Ethics permits Monash Animal Services (MAS) MAS/ARMI/2010/01). Egg cases were collected and tagged with the deposition date and the females were released after 4 to 6 weeks [44]. Tagged eggs were transferred to a closed system (Monash University, Melbourne, Victoria), temperature 14–16.7°C, average 16.8 ± 2.31°C. Embryos were dissected out from the egg cases at regular intervals and euthanized, as above. Specimens were fixed in 4% Paraformaldehyde in Phosphate Buffer Saline (PBS) at 4°C on a rocking platform and then dehydrated into 100% Ethanol or methanol for storage. Embryos were measured and staged following [45].

### Cleared and stained specimens

The specimen were cleared and stained according to a protocol modified from Taylor and Van Dyke [46]. A fixed and dehydrated stage 28 of *C*. *milii* was stained in 0.25 mg/ml Alcian blue in 80% Ethanol and 20% glacial acetic acid for 27.5 hours. The specimen was bleached in 0.45% hydrogen peroxide/0.43% potassium hydroxide in water to remove pigments and cleared in 2.25 mg/ml of trypsin in 30% saturated sodium borate in water. The specimen was cleared in 100%glycerol and imaged on a Leica dissecting microscope with an Axiocam MRM (Zeiss) colour camera.

### Fluorescent immunostained specimens

Whole-mount antibody stainings were performed as described previously [42]. The following mouse monoclonal antibodies were used: Myosin (Mf20) and collagen type II (II-II6B)(from Developmental Studies Hybridoma Bank), Sox9 (marking pre-chondrogenic cartilage and neural crest cells [47, 48]) from Millipore and DAPI (cell nuclei) and visualized using isotype-specific fluorescent Alexa secondary antibodies (Invitrogen). The following fluorophores and colors were used: Cell nuclei: DAPI, white; Myosin (skeletal muscles): Mf20 (488 nm), green; Sox9 (546 nm), red; Collagen type II: 647 nm, blue. Stages 22 to 27 were cleared in 80% glycerol in PBS and mounted on a glass slide. Stages 29 and 30 were embedded in 1% low melting point agarose onto a glass petri dish and cleared in 1:2 Benzylalcohol: Benzyl Benzoate. Whole mounts of stages 22, 25 and 27 were imaged on an inverted Leica SP-5 Confocal microscope with a 20x glycerol objective, stage 23 was imaged on an inverted Leica SP -8 confocal microscope with a 10X dry objective and stages 29 and 30 were imaged on an upright Leica SP-8 Confocal microscope with a 5X dry objective. The stacks were analyzed with Imaris 8 imaging suite and FIJI (Image J). Final image processing was done using Photoshop CS6.

### Holocephali, *Callorhinchus milii*: adults

A synarcual was dissected from an adult *Callorhinchus milii*, defleshed and CT-scanned (X-Tek HMX ST CT scanner, Image and Analysis Centre, Natural History Museum, London, and rendered using the programs Drishti (https://github.com/AjayLimaye/drishti) and Avizo (www.fei.com/software/avizo3d/).

## Results

### Placodermi: *Campbellodus decipiens* (WAM 11.9.1, Fig 1A and 1B)

**Fig 2:**
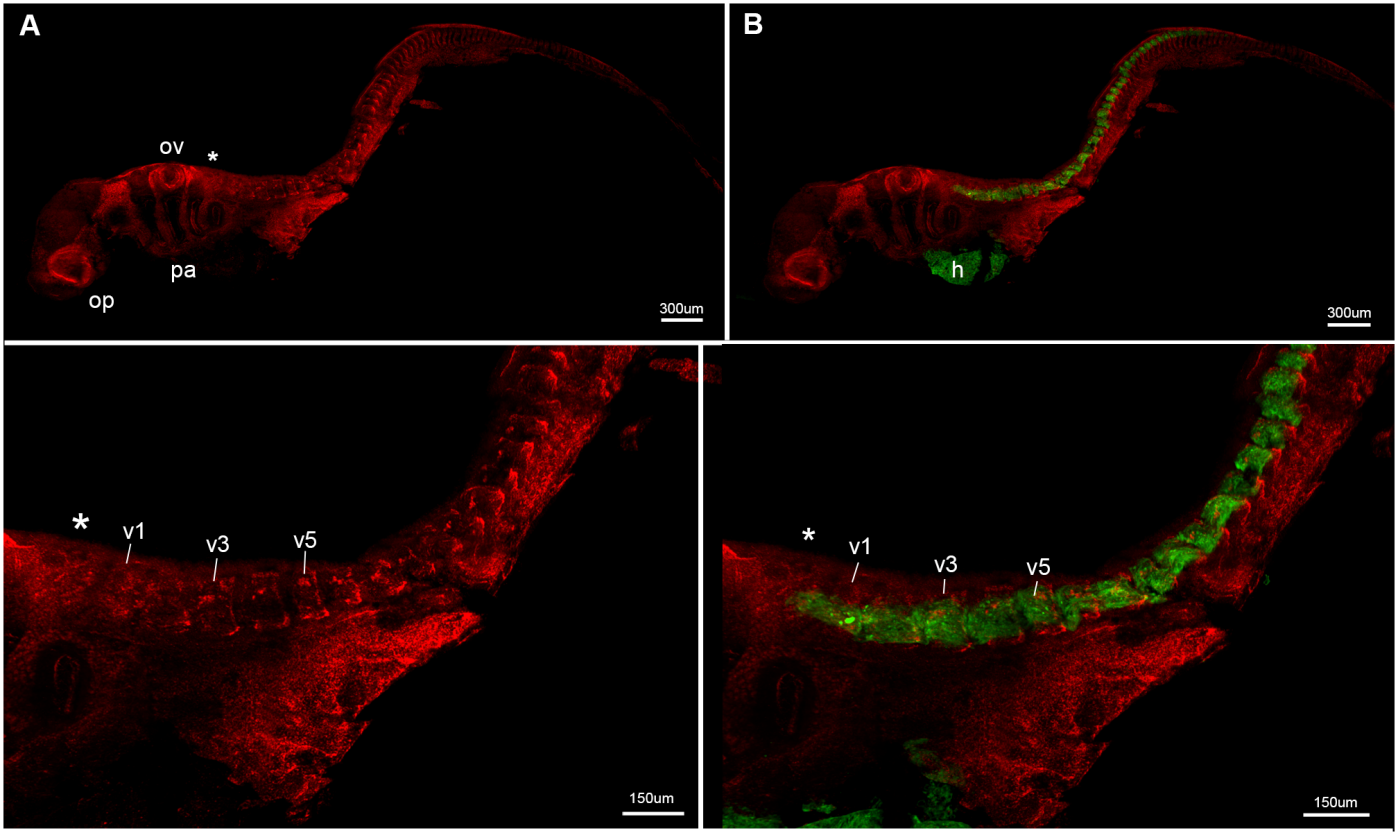
*Callorhinchus milii*, stage 22. **A-D**, lateral view showing general morphology as well as Sox9 (neural crest cells, prechondrogenic cartilage, red) and Mf20 (muscle fibres, developing skeletal musculature, green) staining. Developing vertebrae are separate and distinct at this early ontogenetic stage. Asterisk indicates rear of developing braincase. Abbreviations: h, heart; op, optic capsule; ov, otic vesicle; pa, pharyngeal arches, v1, 3, 5, differentiating vertebrae 1, 3, 5.

The Placodermi are an extinct group of early jawed vertebrates, with current analyses indicating they are paraphyletic, with different taxa resolved to consecutive nodes at the base of the jawed vertebrates (e.g., [49], and references within). Among placoderms, a synarcual has been described from the Ptyctodontida, Arthrodira and Rhenanida [24–26, 50–52]. New high-resolution synchrotron scans of the ptyctodont *Campbellodus decipiens* support previous suggestions that the placoderm synarcual forms from the incorporation of regularly developed vertebral elements (e.g., [24]: figure 2f, g). The synarcual itself is composed of three neural spines, separated by elongate openings for the passage of spinal nerves (sp.n). Immediately posterior to the synarcual are independent neural spines, with flared and rounded bases (representing neural arch bases, larger white arrowhead), that would have rested on the notochord. There is a distinct and sharp ridge running along the rostral margin of the neural spine (small arrowheads), while the dorsal portion of the neural spine is also flared (also [24]: figure 2d). The dorsal part of the synarcual is large and expanded; internally and externally, growth lines are visible, suggesting ongoing cartilage deposition, accompanied by corresponding perichondral ossification externally (Fig 1A and 1B, smaller white arrows). Of the three incorporated vertebral elements, the most rostral is the largest, with subsequent elements decreasing in size caudally, with the third being of similar size to the independent, separate neural elements. These three elements incorporated into the synarcual preserve the flared bases of more caudal vertebrae (asterisk), as well as the ridge along the rostral margin (small arrowheads). However, along with an increase in size, this ridge becomes less distinct in the more rostral vertebra of the synarcual. These differences suggest that the three vertebral elements were added or incorporated successively to the synarcual, rather than at the same time, and were modified during this time. This interpretation is further supported by observations in other ptyctodonts such as *Materpiscis attenboroughi*, where previously independent vertebral elements are being added and incorporated into the rear of the existing synarcual ([24]: figure 2f, g). There is some variation in the numbers of vertebrae being added, with five in *Materpiscis* and four in *Austroptyctodus gardineri*, again suggesting successive and ongoing addition of vertebrae.

### Chondrichthyes; Holocephali: *Callorhinchus milii*

#### Stage 22 (Fig 2)

In stage 22, individual vertebral elements are present, extending posteriorly from the otic vesicle (Fig 2A and 2B). The specimen is stained for Sox9 (red) and Mf20 (green); the former marks pre-chondrogenic cartilage and neural crest cells, including crest cells migrating into the pharyngeal arches (pa; Fig 2A-2C), while the latter indicates developing skeletal musculature and heart (h) (Fig 2B and 2D). Pre-chondrogenic cartilage can be observed dorsally and ventrally in the individual elements more posteriorly, and throughout the elements just behind the otic vesicle and developing braincase (Fig 2C, ov, asterisk, v1-3). The presence of the newly formed fibers of skeletal muscle and the prechondrogenic cartilage indicates that the somites have differentiated by this stage into the myotome and vertebral elements medially, in each individual element. This indicates normal somite development and maturation into the sclerotome and dermomyotome. Individual, differentiated elements (or vertebrae) can be recognized just posterior to the braincase (Fig 2C and 2D, v1-v5), with muscle associated with these, extending from the first differentiating vertebra (Fig 2C, v1) to the developing cartilage at the rear of the braincase.

#### Stage 23 (Fig 3)

**Fig 3:**
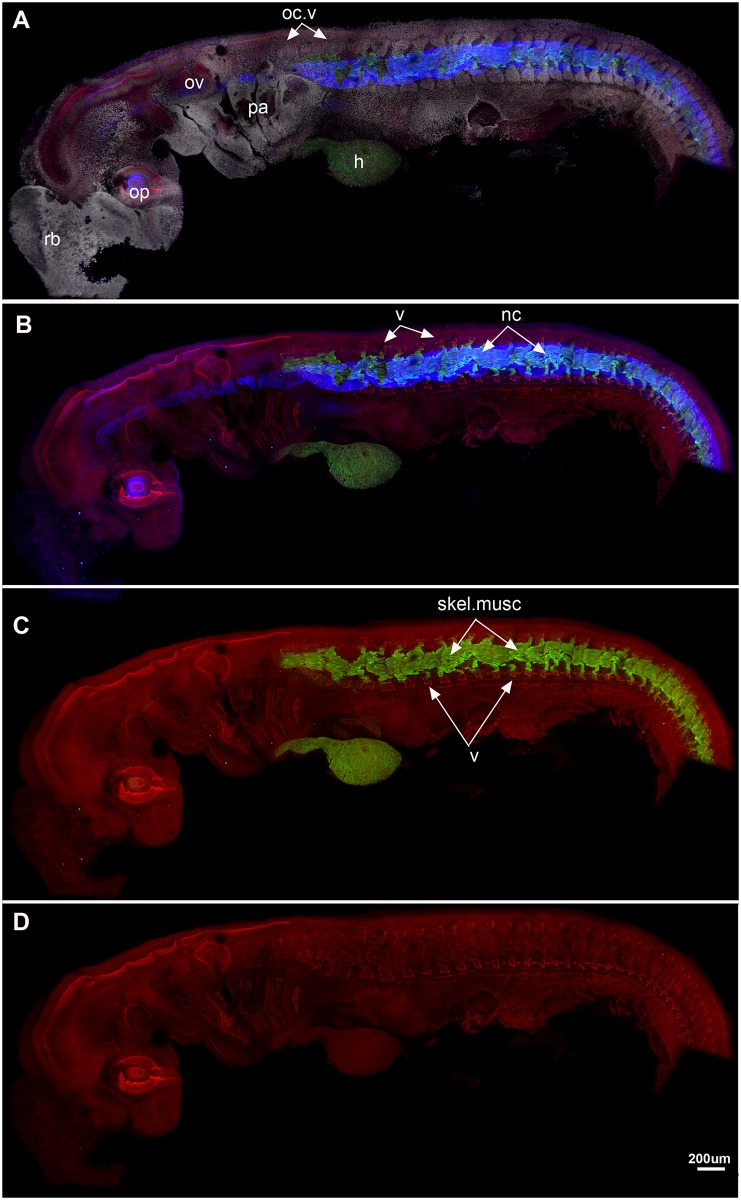
*Callorhinchus milii*, stage 23. A-D, lateral view showing A, staining as in Fig 2, also DAPI (cell nuclei, white) staining. Blue colour indicates collagen type II staining in the notochord. B, As in A, but DAPI staining not visualised; C, Sox9 and Mf20 staining, showing dorsal and ventral vertebral elements (neural and haemal arches) and skeletal musculature; D, Sox9 staining alone. All vertebral elements separate and distinct at this stage. Abbreviations as in Fig 1, also: nc, notochord; oc.v, occipital vertebrae; skel.musc, skeletal musculature; rb, rostral bulb [28].

Stage 23 is characterised by the development of four pharyngeal arches and a large rostral bulb (rb; Fig 3A). The optic cup (op) and otic vesicle (ov) are well developed. The notochord is labelled by collagen type II, clearly marked in blue (Fig 3B) and it extends anteroposteriorly in association with the developing vertebral elements. The trunk musculature has differentiated and spans each vertebrae and, as in Stage 22, is restricted to the middle of these elements (Fig 3C). Individual differentiated vertebrae are still present at this later stage, indicated by the Sox9 staining of the dorsal and ventral prechondrogenic cartilage (Fig 3D) and DAPI staining of cell nuclei (Fig 3A). This indicates that the vertebrae are developing distinct dorsal and ventral neural elements, notably, the most anterior vertebrae have to change shape, and the distance between them appears smaller than in the previous stage. These are identified as the occipital vertebrae, and will eventually fuse to the rear of the braincase (oc.v).

#### Stage 25 (Fig 4A and 4B)

**Fig 4:**
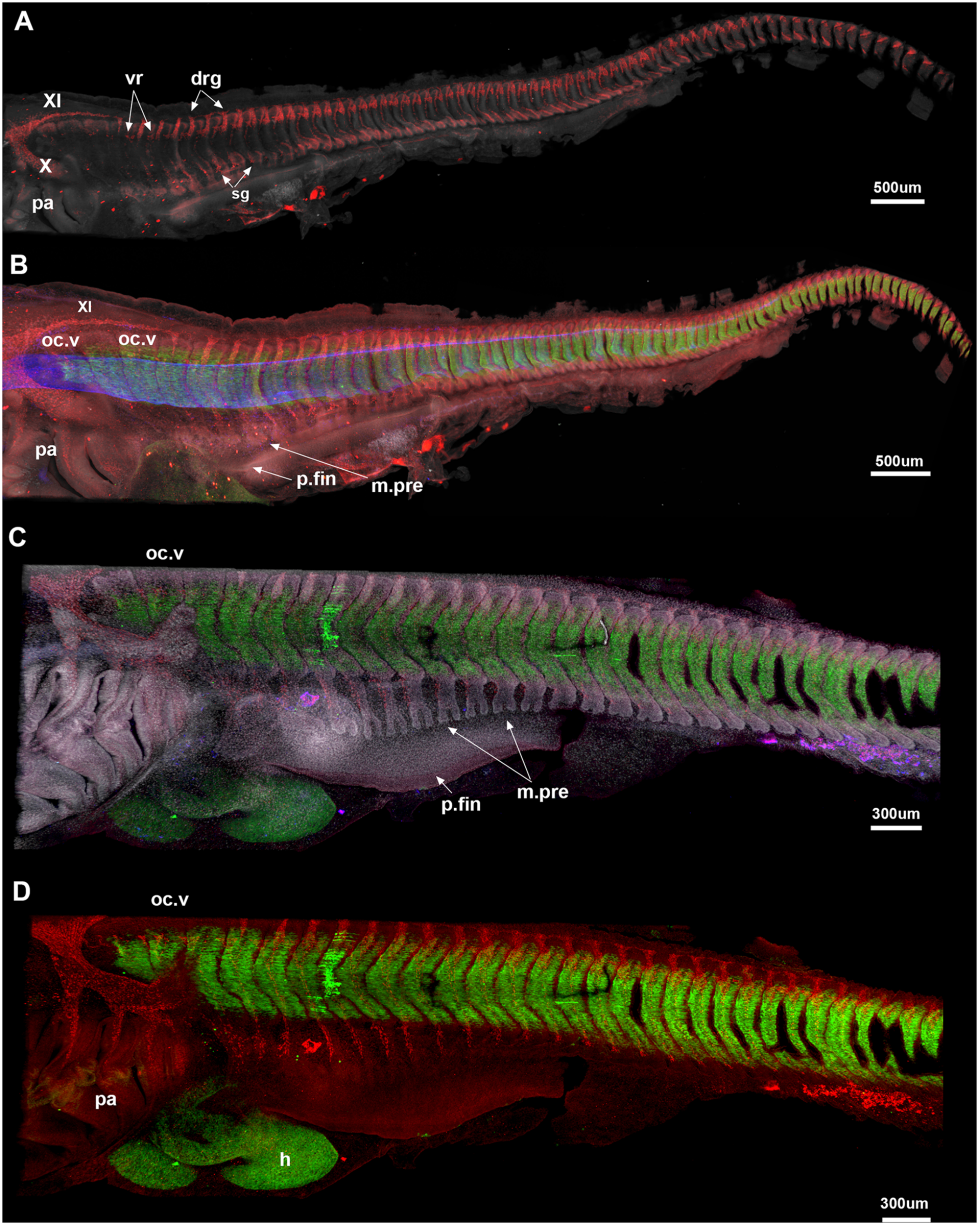
A-E, *Callorhinchus milii*, stages 25 (A, B), 27 (C, D). A, Cell nuclei (white). Sox9 (red) marks dorsal and ventral vertebral elements and neural crest cells; the vagus and accessory nerves (X and XI) are clearly visible. Three anteriormost occipital vertebrae (oc.v) lack the spinal nerves associated with more posterior vertebrae; they appear closer together differ in shape relative to the more posterior vertebrae, which are better developed and still distinct from each other. B, notochord (blue) and developing skeletal musculature are visualized. The pectoral fin bud is beginning to develop (p.fin), while the pectoral fin musculature is differentiating from the ventral myotome (m.pre). C, Stage 27, Sox9 no longer stains the vertebral elements (differentiated beyond the prechondrogenic cartilage stage); they are best seen via DAPI staining (cell nuclei). All vertebral elements are still separate from one another. Developing pectoral fin musculature is more distinctly bifid in shape. Abbreviations as in previous figures, also: drg, dorsal root ganglia; m.pre, pectoral fin muscle precursor; p.fin, pectoral fin; sg, sympathetic ganglia; vr, ventral root.

Dorsal and ventral prechondrogenic cartilage of the vertebrae continues to develop (Fig 4A), but sox9 (red) also marks neural crest cells as they migrate ventrally into the pharyngeal arches, and caudally to form the vagus and accessory nerves (X, XI), as well as components of the spinal nerves, including the dorsal roots and ganglia and the ventral roots and sympathetic ganglia ventrally. The three vertebrae (oc.v, Fig 4A and 4B) are ventral to the developing accessory nerve, and caudal to the vagus nerve, representing the occipital vertebrae (Fig 4B, oc.v); these will contribute to the occiput in later ontogeny, as noted above. There is some indication of ventral roots developing in the occipital vertebrae, but the dorsal root ganglia are absent from these three vertebrae. The spinal nerves run dorsoventrally across the vertebrae including the occipital anteroventrally, just posterior to the pharyngeal arches, DAPI marks a bifid protrusion associated with each myotome, better developed in later stages as described below, again representing developing fin musculature. Mf20 (green, Fig 4B) marks the axial skeletal musculature associated with the middle of each differentiated vertebra. All vertebrae are separate and developing normally but as noted in stage 23, the occipital vertebrae are changing morphology and appear closer to each other in this stage.

#### Stage 27 (Fig 4C and 4D)

The vertebrae in stage 27 are no longer expressing Sox9 (no longer at the pre-chondrogenic stage) but are better visualised through a DAPI staining (Fig 4C). As in earlier stages, the more anterior occipital vertebrae appear closer to each other, while the posterior vertebral elements remain more clearly separated. Skeletal muscle is well differentiated and remains closely associated with each vertebra, including the occipital vertebrae. DAPI staining marks the series of muscle progenitors associated with the developing pectoral fin, which now have strongly bifid ventral edges, as they continue to migrate into the fin bud (m.pre, Fig 4C). As noted, these first appear in stage 25, although their bifid nature is less apparent and the fin bud itself is smaller (e.g., Fig 3B). The ventral nerve roots in the occipital vertebrae are better-developed, although the dorsal root ganglia are still absent (Fig 4D).

### Stage 28 (Fig 5A-5C)

**Fig 5:**
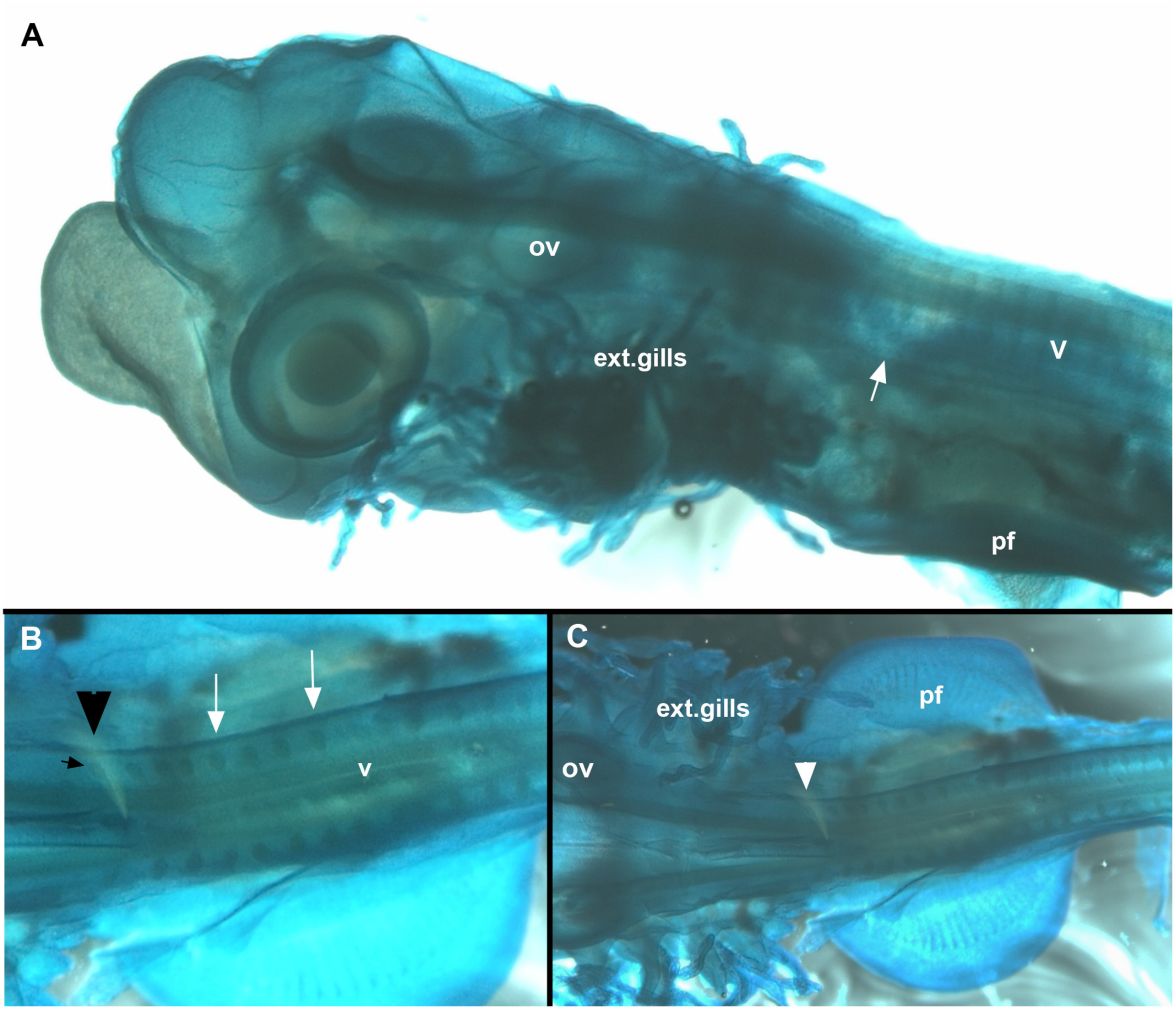
*Callorhinchus milii*, stage 28, A-C. Cleared and stained specimen (Alcian blue). A, lateral view, showing embryo with external gills and developing vertebrae. White arrow indicates anterior vertebrae that have become misshapen, indicating fusion and coalescence. The position of these relative to the pharyngeal arches and pectoral fin indicates that these are fusing to the occipital region of the braincase, rather than as part of a more posterior synarcual. More posterior vertebrae appear normal at this stage. B, C, dorsal view, arrowhead indicates occipital anteriorly and vertebrae posteriorly. White arrow indicates foramina Abbreviations, as in previous figures, also: ext.gills, external gills.

A specimen cleared and stained with Alcian blue (cartilage) show the posterior edge of the skull (with occipital vertebrae being incorporated, Fig 5B and 5C, arrowheads) as well as more posterior elements of the vertebral column (Fig 5A-5C, v). Separate vertebral elements can still be recognized in both lateral and ventral views, with the edges of individual vertebrae still visible, along with large, open nerve foramina (Fig 5B, white arrows). In Fig 5A, these more posterior vertebral elements are narrow and regular in shape; however, more anteriorly, the elements are more irregular in shape (on either side of white arrow), providing some indication that fusion of cartilaginous vertebral elements has begun by this stage. With respect to later stages described below, these would appear to be fused to the occipital region.

#### Stage 29 (Fig 6A and 6B)

**Fig 6:**
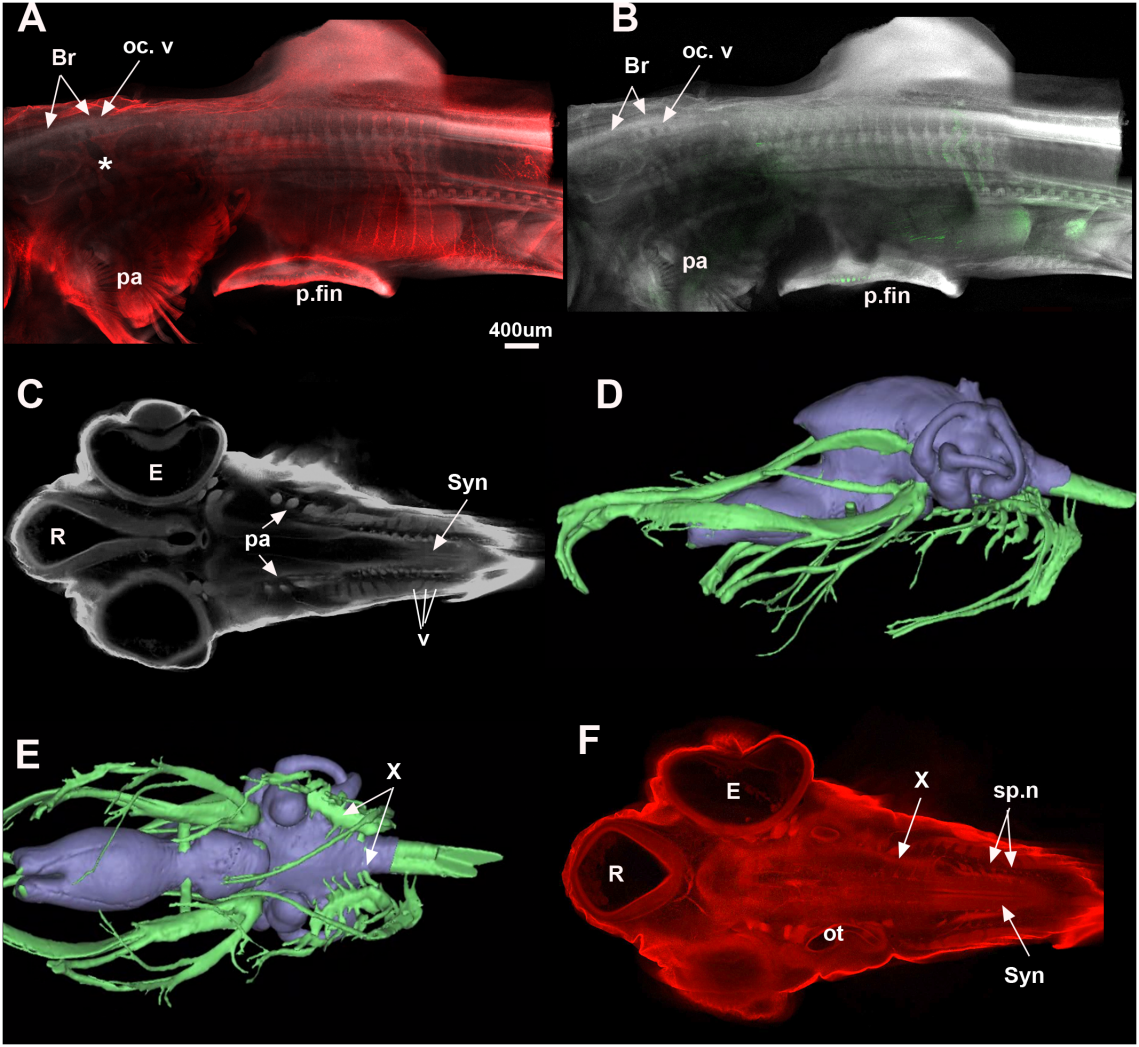
*Callorhinchus milii*. A, B, Stage 29 (maximum projection, Sox9 red, DAPI white, Mf20 green) shows that the occipital vertebrae are still distinct from each other but that the braincase is now well developed. The posterior elements are still separate but show distinct dorsal and ventral elements for each vertebra. In stage 30 (C, F; one plane in Z, Sox9 red, DAPI white), the synarcual has started to form and can be recognised medially. The individual vertebral elements are still visible laterally (v). The vagus and accessory nerves are visible lateral to the mineralised braincase (C). D, E show distribution of the cranial nerves in a *C*. *milii* hatchling (Khonsari et al. 2013). Abbreviations as in previous figures, also Br, Braincase; E, Eye; R, Rostrum; Syn, synarcual. Images in Fig 6D and 6E taken as screenshots from a 3-D reconstructed CT scan model; Wikimedia Commons datafile, Khonsari et al. 2013. BMC Biology. doi:10.1186/1741-7007-11-27.

The vertebrae are still individual elements posterior to the braincase, although fusion and coalescence is observed anteriorly (Fig 6A and 6B, oc.v). The position of this fusion dorsal to the pharyngeal arches (pa) and posterior cranial nerves (Fig 6A, asterisk) suggest that this is occurring with respect to the occipital region of the braincase, rather than within the separate synarcual (compare Fig 6A and 6B with reconstruction of late stage embryo of *Callorhinchus milii*, Fig 6D and 6E [53]). Vertebral elements have stopped expressing Sox9, showing that they are further along in the chondrogenic programme. Few skeletal muscle fibres are stained in green as Mf20 only stains newly formed fibres. However, it shows that the pectoral fin musculature is newly differentiated (Fig 6B).

#### Stage 30 (Fig 6C and 6F)

Z sections through a confocal stack of a dorsally mounted specimen. A more ventral Z plane shows the synarcual forming posterior to the pharyngeal arches (pa, syn, Fig 6C) with the ventral elements of the vertebrae still visible in lateral view (v, Fig 6C). A more dorsal plane in Z (Fig 6F) shows the branches of the X nerve anteriorly (X), also marking the position of the pharyngeal region (Fig 6D and 6E) relative to the otic capsule and synarcual. Spino-occipital nerves can be seen in association with the synarcual (sp.n, syn, Fig 6F).

#### Adult (Fig 7)

**Fig 7:**
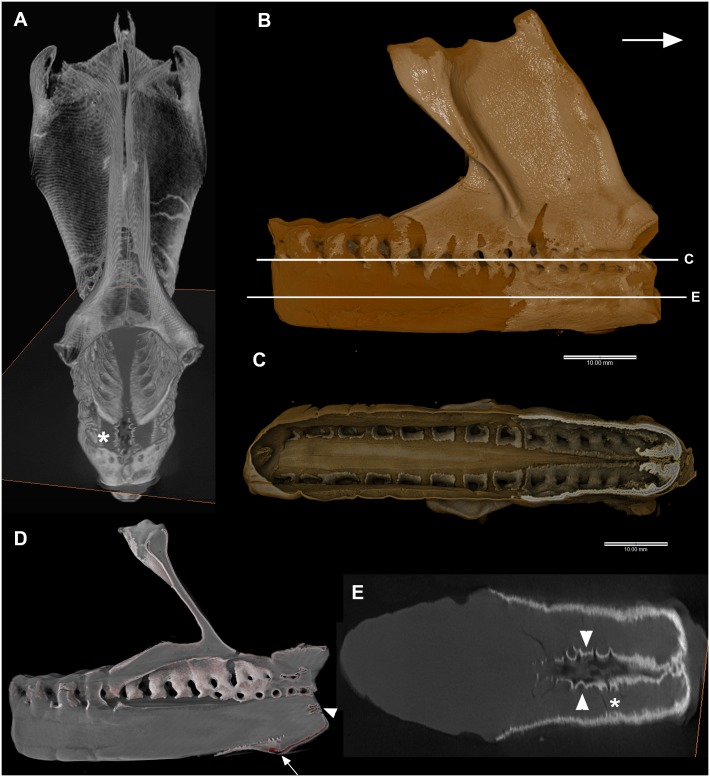
A-E, *Callorhinchus milii*, adult, A-E. A, 3D volume rendering (Aviso), anterior view. Asterisk indicates comparable region in 7E. B, 3D volume rendering, lateral view (Drishti), arrow indicates anterior. False color differences indicate surficial mineralization (yellow) and unmineralized cartilage (brown). Levels of Fig 7C and 7E indicated. Note that mineralization proceeds internally via the spinal nerve foramina (C, virtual section, Drishti). D, virtual section (Drishti) showing pairs of spinal foramina anteriorly, continuous foramina posterior. Arrowhead indicates region in Fig 7E, arrow indicates ventral area where vertebral elements have not fully fused or coalesced. E, virtual section (Drishti) through ventral part of synarcual showing unfused elements (arrowheads) that have become mineralized, preventing further fusion and preserving a portion of the original shape.

In an adult individual, the synarcual has developed an extensive dorsal keel, vertebrae have become fused into a single unit, and mineralization of the cartilage is occurring. Rendered CT-scans and virtual sections clearly show that this mineralization extends rostrocaudally, dorsoventally (both, Fig 7B), and from external to internal. In Fig 7C and 7D, virtual sections through the synarcual show how the mineralization extends through the nerve foramina on both sides of the synarcual, meeting in the midline. Fig 7E represents a virtual slice through the ventral portion of the synarcual, below the spinal nerve foramina. The asterisk in Fig 7E marks a region of the synarcual also visible in Fig 7A (anterior view). Patterns of mineralization (Fig 7E, lighter areas representing high density regions) again are clearly rostro-caudal, but also show that ventralmost fusion of the vertebral elements is incomplete, and the mineralization is occurring along two separate halves of the synarcual internally (also Fig 7A and 7D, white arrow). The mineralization continues internally along the midline, with some indication of the retention of individual ventral elements (Fig 7E, arrowheads). The white arrowhead in Fig 7C and 7D shows another small area of mineralization more dorsally, again representing incomplete fusion or inclusion of the ventral vertebral elements, but at the anterior margin of the synarcual.

## Discussion

The process of somite segmentation during early development is well understood, with presomitic mesoderm under the influence of two opposing gradients (retinoic acid rostro-caudally; *Fgf* caudo-rostrally) and the oscillating segmentation clock, itself controlled by genes such as *Notch*, *Fgf* and *Wnt* [6, 10]. Somite pairs form rostro-caudally, and subsequently differentiate to form the sclerotome ventrally and the dermomyotome dorsally. The sclerotome contributes to the axial skeleton, and the dermomyotome to the dorsal epithelium and musculature. Segmentation is well understood in zebrafish [54] and amniotes, including a process known as resegmentation in the latter, where the sclerotome becomes polarized into distinct rostral and caudal halves. During resegmentation, caudal and rostral halves fuse, to form a single vertebra. Associated muscles do not undergo resegmentation, and so remain out of register, but functional, with respect to the vertebrae. Resegmentation was thought to be related to the size of the sclerotome, which is large in amniotes, but much smaller in anamniotes (e.g., amphibians, fish). Scaal and Wiegreffe ([55]: figure 4) review differences between the amniote and anamniotes, including the relatively smaller sclerotome differentiating at the medioventral corner of the somite and the larger myotome in the latter. They also note that in anamniotes, the myotome is the first to differentiate. Resegmentation has been recently demonstrated in the amphibians, in the axolotl [56], but has not been fully investigated in chondrichthyans or fishes in general, except for the zebrafish [57].

Observations in the Holocephali indicate that somite segmentation occurs normally, because in early ontogenetic stages (st 23–27), separate and distinct vertebrae are developing, indicated by the presence of prechondrogenic cartilage dorsally and ventrally (neural and haemal elements), with associated muscle fibres (Figs 2 and 3). In later stages, these developing (differentiating) vertebrae are associated with the spinal nerves (red, Fig 5A, 5B and 5D). Only ventral roots are present in the first three occipital somites, as previously described in the chick and mammals [58], and chondrichthyans [59]. The first dorsal root ganglion appears in more caudal vertebrae, along with the ventral roots, these meeting to form the spinal nerve crossing the vertebra (Fig 5). The dorsal and ventral roots innervate the epaxial and hypaxial musculature, respectively. V-shaped muscles are present, associated with each vertebra. Pectoral fin muscles are developing, as direct bifid extensions of the ventral (hypaxial) region of the musculature into the fin, as observed in other chondrichthyans (*Scyliorhinus*, catshark [59–61]). At these stages, it is difficult to see a developing sclerotome, which, if following the anamniote pattern, would be represented by a small cluster of medioventral cells, and so difficult to see in lateral view (Figs 2–5). However, the dominance of the myotomal muscle derivatives corresponds to observations that the myotome differentiates earlier and dominates in anamniotes [55].

However, we cannot observe whether resegmentation occurs in *Callorhinchus*; the presence of resegmentation in amphibians [56], and ‘leaky’ resegmentation in zebrafish [57] suggests that resegmentation occurs, phylogenetically, in the group Osteichthyes (bony fishes, including tetrapods). Chondrichthyes, including *Callorhinchus* (Holocephali) and sharks such as *Scyliorhinus* (Elasmobranchii) form the sister group to the Osteichthyes, so resegmenation will have to be investigated further to establish this process as a general character for jawed vertebrates.

Nevertheless, the developing vertebrae and skeletal musculature indicate that segmentation has proceeded normally in *Callorhinchus*, with differentiation of the somite into sclerotome and dermomyotome, leading to the normal development of the vertebrae and associated musculature. Our observations of stages 23–30 suggest that to this point, fusion occurs with respect to vertebrae that are dorsal to the branchial arches, and more posterior cranial nerves, posterior to the otic capsule and anterior to the pectoral fin (Fig 6). We suggest that this represents fusion of vertebral elements to the occipital region of the braincase, which is located in this relative position (cf. reconstructions of *Callorhinchus* embryo (Fig 6D and 6E, [53]). The synarcual, as a fusion of the anteriormost vertebrate into a single structure, develops subsequently from the more posterior vertebrae, with the incorporation of vertebrae on an ongoing basis [24]. This is similar to previous observations that in batoid chondrichthyans, the synarcual first forms from separate vertebral centres [27, 29]. In the batoids, Claeson [27] noted that early in ontogeny, the synarcual formed from coalescing vertebral chondrification centres, and was subsequently covered by a prismatic cartilage layer. In *Callorhinchus*, mineralization of the synarcual occurs as the prismatic layer extends caudally and ventrally, and internally via the nerve foramina (Fig 7). In the anteroventral part of the synarcual, mineralization outlines individual vertebrae (Fig 7D and 7E), supporting the suggestion above that the vertebrae first develop normally and are only later incorporated into the synarcual. Mineralization of the cartilage, as a surficial prismatic layer (e.g., Fig 7C), prevented any further incorporation of these vertebrae, allowing them to still be recognized within the synarcual.

The presence of individual and distinct vertebral elements within the synarcual is an important criterion for distinguishing between vertebral fusion due to irregularities in somite formation (individual vertebrae would not have formed), versus fusion later in ontogeny. In the latter, somites and vertebrae develop normally before fusion occurs and can be recognised. This is relevant to extant taxa, but also fossil forms. In the placoderm synarcual, although ontogenies cannot be directly observed in fossil taxa, perichondral ossification of these elements can ‘freeze’ ontogenies and provide evidence for their normal development and subsequent incorporation into the synarcual. Taxa such as the arthrodires *Cowralepis* and *Compagopiscis*, and the ptyctodont *Materpiscis* preserve distinct vertebral morphologies within the synarcual (distinct rounded arch bases) or the addition of elements to the synarcual ([24]: figures 2f, g and 4). By comparison, the vertebral elements in the ptyctodont *Campbellodus* retain some evidence of individuality (flared, rounded bases, rostral ridges), but there has been notable modification of these arches as they were incorporated into the synarcual, including a flared dorsal keel (Fig 1). Modification is greatest anteriorly, indicating an ongoing incorporation of vertebral elements, as in *Callorhinchus*. In *Austroptyctodus* the individual vertebral elements that make up the synarcual cannot be identified, although the rounded bases of the neural arches can still be distinguished ([24]: figure 2e).

Clues as to how originally separate and distinct vertebral elements could be added to and incorporated into the synarcual are provided by recent investigations into teleosts such as the salmon (Osteichthyes); these have been intensively studied for defects in the vertebral column, including fusion, caused by environmental factors such as temperature changes and stress. Tissues associated with the vertebral column including bone, cartilage and intervertebral tissues (notochord) appear highly plastic under these conditions. For example, Witten et al. [16, 17, 14, 62] found that vertebrae developed normally, but response to stresses included a transformation of intervertebral tissues into cartilage and subsequently bone, while osteoblast (bone-depositing) cells in the vertebral growth zones underwent a metaplastic change to become chondroblasts (cartilage-depositing) cells, depositing cartilage between the vertebrae (also [18, 19, 63]). Genes associated with this metaplastic cell transformation during vertebral fusion include *matrillin-1* [64] and *sox9* [18].

These observations, in a range of vertebrates, suggest that the synarcual resulted from a transformation of cells and tissues located between vertebrae, rather than a failure of somite segmentation. Among human vertebral disorders, this is more comparable to conditions such as Fibrodysplasia Ossificans Progressiva (FOP), which involves a metamorphosis of soft connective and muscle tissue associated with the vertebrae into cartilage and endochondral bone, resulting in fusion (e.g., [38, 39]). Genes involved in this process include the bone morphogenetic proteins (*Bmp*) and their receptors, and *noggin* (reviewed in [40]; [65]). The transformation into endochondral bone in FOP involves a cartilaginous precursor stage, which is related to a gain-of-function mutation in certain *Bmp* receptors, resulting in increased chondrogenic differentiation and thus increased bone formation, to fuse together existing vertebrae (e.g., [65]). As well, Dittman et al. [66] noted that inactivation of inhibitory Hedgehog family receptor *patched1* resulted in chondrocyte proliferation in the intervertebral discs and fusion of vertebrae.

Metamorphic and transformative processes similar to those involved in FOP and fusion of teleost vertebrae, affecting initially normally developed vertebrae, could be envisaged for the placoderm and chondrichthyan synarcual (*Callorhinchus* and the batoid rays), specifically with respect to the intervertebral tissues, connective tissues and muscle, and the transformation of these into extra cartilage. Placoderms and chondrichthyans lack the capacity to develop endochondral bone and instead mineralization is surficial; in placoderms, heterotopic perichondral bone is formed, and in chondrichthyans, heterotopic prismatic cartilage is formed as a thin layer on the cartilaginous surface.

Teleosts and chondrichthyans present an opportunity to study genes involved in the fusion of vertebrae in considerable detail, while chondrichthyans allow a particular focus on the cervical region. Candidate genes related to spinal fusion in teleosts have been identified, but fusion depends on environmental conditions generating animal stress; by comparison, fusion of vertebrae into a synarcual occurs normally as part of development in chondrichthyans such as *Callorhinchus* and the batoids, and consistently in the anterior part of the vertebral column. This makes these chondrichthyans more suitable as ‘evolutionary mutant models’ (see Table 1 in [42], also [43]) for studying genetic/developmental mechanisms resulting in spinal fusion in humans, as distinct from trauma/disease processes that cause this fusion. Chondrichthyans possessing synarcuals such as *Callorhinchus* and *Leucoraja* (Little skate) are becoming new model animals in genetic studies, with a genome now available for both taxa [67, 68]. Future work should focus on further identifying genes involved in synarcual development in these taxa, to provide a framework for future medical research into human vertebral disease syndromes.
